# High prevalence of epilepsy in Northern Rwanda: Exploring gender differences

**DOI:** 10.1002/brb3.2377

**Published:** 2021-10-17

**Authors:** Peter Dedeken, Fidele Sebera, Sylvestre Mutungirehe, Ieme Garrez, Josiane Umwiringirwa, Frank Van Steenkiste, Paul A. J. M. Boon, Dirk E. Teuwen

**Affiliations:** ^1^ Department of Neurology Ghent University Hospital Ghent Belgium; ^2^ Department of Corporate Societal Responsibility UCB Pharma Brussels Belgium; ^3^ Department of Neurology Heilig Hart Ziekenhuis Lier Belgium; ^4^ Department of Neurology, Ndera CARAES Neuro‐psychiatric Hospital Kigali Rwanda; ^5^ Centre Hospitalier Universitaire Kigali Kigali Rwanda; ^6^ Department of Neurology National University Hospital FANN University of Cheikh Anta Diop Dakar Senegal; ^7^ 4Brain Ghent Belgium; ^8^ Psychiatric Center Sint‐Jan‐Baptist Zelzate Belgium

**Keywords:** door‐to‐door, epilepsy, gender gap, prevalence

## Abstract

**Introduction:**

In sub‐Saharan Africa (SSA), the prevalence of lifetime epilepsy varies widely between subregions and is higher in rural compared to urban regions. Observed versus expected numbers of patients with epilepsy (PwE) in the northern province of Rwanda did not match the prevalence of 49‰ reported in 2005 in Rwanda. We report a confirmatory prevalence study focused on gender‐specific observations.

**Methods:**

A cross‐sectional door‐to‐door approach was used in three rural villages. First, epilepsy screening using the Kinyarwanda version of the Limoges questionnaire was performed. Second, confirmation of epilepsy diagnosis was completed by trained physicians.

**Results:**

In total, 2681 persons (56.14% female) were screened. Of 168 positively screened, 128 persons were diagnosed with epilepsy confirming the prevalence of lifetime epilepsy of 47.7‰ (CI 39.8–56.8). The diagnosis gap was 62.5% with 80 newly diagnosed. The overall female:male ratio was 1.61:1.00. A male preponderance below 9 years of age inverted to a female preponderance above 20 years of age. Female PwE had an older age at first seizure, reported different reasons for not seeking care, and differed from male PwE in possible etiology. For previously diagnosed PwE, the treatment gap was more than 77%.

**Conclusion:**

A high prevalence in rural areas was confirmed, with an observed female/male ratio among the highest of published door‐to‐door surveys in SSA. Gender differences in associated co‐morbidities and age at first seizure warrant future research of underlying etiologies and possible survival bias. A better understanding and focus on gender‐associated care‐seeking patterns, education, and specific needs are recommended.

## Abstract 2

We confirmed the high prevalence of epilepsy in Rwanda and observed gender differences. Of 2681 screened persons, 168 were positively screened and 128 were diagnosed with epilepsy resulting in an epilepsy prevalence of 47.7‰. Female patients with epilepsy had an older age at first seizure and reported different reasons for not seeking care. This results in a need for further epilepsy etiology research and informs on gender‐specific needs for public health programs.

## 1 INTRODUCTION

Epilepsy is characterized by unpredictable, recurrent seizures and may have cognitive, psychological, and socio‐economic consequences (Fisher, Cross, French, et al., 2017). This common chronic disease is affecting over 70 million patients living with epilepsy (PwE) worldwide, of whom nearly 85% live in low‐income and low‐ and middle‐income countries (LLMIC) (Ngugi et al., 2010). It contributes nearly to 1% in the global burden of disease and 20% of the global burden of epilepsy measured in disability‐adjusted life years (DALYs) in sub‐Saharan Africa (SSA) (GBD 2015 Neurological Disorders Collaborator Group, 2017).

The estimated prevalence of epilepsy varies widely between high‐income counties (range 4 to 7‰) and LLMIC (range 5 to 74‰) (Ngugi et al., 2010; Preux & Druet‐Cabanac, 2005). A recent meta‐analysis of 38 studies in 19 SSA countries estimated an overall prevalence of active and lifetime epilepsy at 9‰ and 16‰ respectively, varying widely between subregions but with a consistent trend of higher epilepsy prevalence in rural compared to urban settings (Owolabi et al., 2020). The epilepsy prevalence in Rwanda was estimated at 49.3‰ in a community‐based door‐to‐door survey in 2005, also indicating higher prevalence in rural compared to urban areas (Sebera et al., 2015).

Rwanda is a landlocked country in East Africa and home to more than 12.5 million inhabitants, with a mean age of 20 y, of which less than 20% live in urban areas (Nyirandagijimana et al., 2017). A community‐based health insurance model provides cost coverage. Mental health care in Rwanda, including epilepsy care, has a leveled pyramidal structure, ensuring decentralization in all health facilities, ranging from primary level health centers under supervision of secondary district or province hospitals to tertiary reference hospitals (Nyirandagijimana et al., 2017). On average in rural areas, health centers provide care to the population of 5.2 sectors, serving in turn 6.9 villages per sector (Rwanda Administration, n.d.). With three neurologists in Rwanda access to neurology care is limited.

During a feasibility assessment for a study on epilepsy and comorbid depression in an urban and a rural setting, the observed number of PwE at health centers in rural areas of the northern province was unexpectedly low compared to our estimates based on a previously reported prevalence of 49‰, triggering a confirmatory study of epilepsy prevalence in rural areas in Rwanda (Sebera et al., 2015).

We report the results of a door‐to‐door survey estimating epilepsy prevalence in three villages of the Musanze Province in Northern Rwanda, using the Limoges epilepsy screening questionnaire (Diagana et al., 2006; Preux et al., 2003).

## 2 MATERIAL AND METHODS

### 2.1 Study area, conduct, and sample size

**FIGURE 1:**
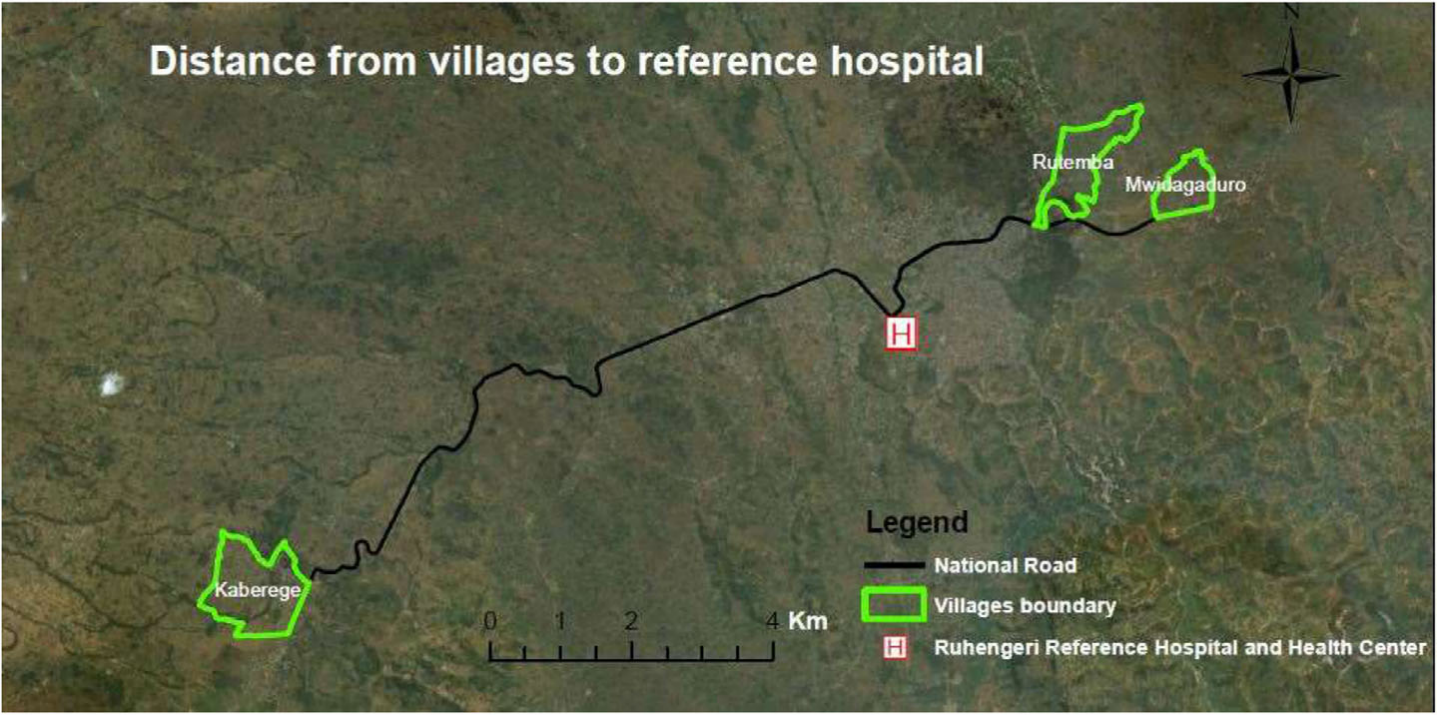
Map study areas and distance to Ruhengeri referral hospital (courtesy: Dr. Yvonne Akimana, Soil and Environmental Management Department, University of Rwanda, 2019)

We conducted a cross‐sectional study with a door‐to‐door approach in three rural villages, that is, Kaberege (supervised by Gataraga Health Center), Mwidagaduro, and Rutemba (supervised Karwasa health center) in the Musanze District (Northern Province, Rwanda). The distance from Kaberege, Mwidagaduro, and Rutemba villages to the Ruhengeri Reference Hospital is 13, 6, and 4 km, respectively (Figure 1). These villages were selected by the directors of the Ruhengeri referral hospital and health centers in agreement with the village elders based on total population, distance to the health centers, rural character, and absence of previous epilepsy awareness campaigns

The low number of PwE observed in the regions called for an assumption on the possible prevalence in order to calculate the required sample size. We used a conservative prevalence of 10‰, in line with another large study in sub‐Saharan Africa, which was only 20% of the previously reported Rwandan prevalence. A sample size of 2000 persons was calculated with a precision of 0.5% and a 97.5% confidence interval (CI) (Kohn & Senyak, 2021; Sebera et al., 2015).

We used the 2017 census data as reference for the total population, provided by the CHW supervisors during preparatory meetings. The census data were not broken down by gender or age groups.

### 2.2 Screening questionnaire and data collection

The Limoges epilepsy screening questionnaire “part 2” was used for screening for epilepsy, which had previously been translated into Kinyarwanda and adapted to the sociocultural context of Rwanda (P.‐M. Preux et al., 2003; Sebera et al., 2015). The questionnaire was initially validated in Mauritania with sensitivity and specificity of 95.1% and 65.6% respectively (Diagana et al., 2006).

We used electronic data capturing, avoiding double data entry and possible paper data loss. An application for mobile devices, Android version 4.0 and above, was built reflecting the questionnaire. Data from the screening questionnaire were collected on a Rwanda‐based server, maintained by One Family Health, a non‐governmental organization based in Kigali, Rwanda.

Data on demographics, clinical characteristics, treatment, and technical investigations of positive screened subjects, were collected in an Excel spreadsheet.

### 2.3 Survey process

The door‐to‐door survey process, involved three phases.

#### Phase I: Preparatory meetings and training

A first preparatory meeting was organized with the Administrative Authority of the Musanze Health District and medical staff of the Ruhengeri Referral Hospital (RHH).

Thereafter, a series of meetings were organized with staff and responsible of the two health centers, elders of the three villages, and supervisor of community health workers (CHWs) in those villages. The supervisor provided data on the number of households and family members. The village elder granted authorization to complete the door‐to‐door survey and allowed CHWs to accompany enumerators. As CHWs are familiar with all villagers, they were engaged to streamline home visits and to ensure screening completeness for each household.

Ten enumerators, recruited among the medically trained nursing staff of the two health centers, were appointed by their respective supervisor. They completed an epilepsy training provided by two project physicians, a Rwandan general practitioner with 4‐year experience in epilepsy, and a French‐speaking senior resident‐neurologist of Ghent University (Ghent, Belgium). Subsequently, the enumerators were trained in the administration of the screening questionnaire, the use of the mobile device, and the project specific application. Enumerators were assigned to screen the village covered by their respective health centers. Rwandan co‐workers were provided the per diem as recommended by the Rwandan Authorities (Official Gazette, 2016).

#### Phase II: Screening through door‐to‐door household visits

Trained enumerators and CHWs were paired in teams of two and visited families at their homes. Household members were explained the survey purpose and possible benefits/risks. A verbal informed consent was obtained and answers to the five questions were recorded electronically. For children, mothers responded to the questions. In the event a family member was absent, CHWs and enumerators planned a return visit. If at the return visit, the family member was not home, he/she was considered as not screened.

#### Phase III: Confirmation of epilepsy diagnosis

Persons who screened positive on one or more questions were accompanied to a neurology consultation at the health center for detailed medical history, medical examination, and in‐depth narrative of seizure description and frequency. The senior resident was assisted by a bilingual nurse with experience in epilepsy as a translator. Caregivers responded for minors and mentally ill persons. Seizure frequency was based on patient reporting. Disclosure of infectious diseases or other conditions was left at the discretion of the subject. A clinical diagnosis of epilepsy, if applicable, was made by the physician. Epilepsy was defined as unprovoked recurrent seizures occurring more than 24 h apart, including active and lifetime epilepsy. In case of doubt, final diagnostic decision was taken in consultation with the neurologist of the Ndera tertiary reference center through a telephone conversation.

### 2.4 Data analysis

Descriptive analysis was performed on anonymized data. Seizure classification according to the 2017 seizure classification was performed by both the senior resident and a general neurologist after review of the available documentation (Fisher, Cross, D'Souza, et al., 2017; Fisher, Cross, French, et al., 2017). In case of discrepancies between physicians, a consensus meeting provided a final outcome. Data were analyzed using Excel functions; *t*‐tests for comparison of means and Chi‐squared test were used for intergroup differences. Prevalence was calculated using sample‐size.net (Kohn & Senyak, 2021).

### 2.5 Ethical review committee and local health and administrative authorities

The study was conducted within the framework of the investigational epilepsy and co‐morbid depression study approved by the College of Medicine and Health Sciences–Institutional Review Board of the University of Rwanda (Kigali, Rwanda) [461/CMBS‐IRB/2016]. Additional approval of the local Administrative Authority of the Musanze District and medical department of the Ruhengeri Reference hospital was obtained.

### 2.6 Literature review

To evaluate the consistency of the observed gender differences, a literature search in Medline, PubMed, French and Portuguese literature on “prevalence,” “epilepsy,” “sub‐Saharan Africa,” and “screening” was performed. Female/male ratios of PwE were tabulated only in case of full manuscripts with a door‐to‐door design and if prevalence by gender was reported.

## 3 RESULTS

### 3.1 Prevalence and diagnosis gap

The study was conducted in September and October 2017.

In total, 2681 (79.5%) out of 3372 persons total population, from 806 households were screened. Of the screened villagers, 56.14% were female. A total of 168 persons replied positively to at least one question and 128 persons were diagnosed with epilepsy by the physicians, yielding a prevalence of lifetime epilepsy of 47.7‰ (CI 39.8–56.8‰). Village specific prevalence were 36.2, 63.4, and 48.4‰ for the sites of Kabarege, Mwidagaduro, and Rutemba respectively, with overlapping CIs (Table 1). With a positive predictive value of 76% (128/168), the Limoges questionnaire performed well. Of all confirmed cases, 48 (37.5%) were previously diagnosed PwE, resulting in a diagnosis gap of 62.5%.

**TABLE 1:** Screening and crude epilepsy prevalence by village

**Village**	**Kaberege**	**Mwidagaduro**	**Rutemba**	**Total**
Health center	Gataraga	Karwasa	Karwasa	
Population				
Households (*n*)	337	222	247	806
Citizens per census (*n*)	1307	954	1111	3372
Men [*n* (%)]	622 (47.6)	438 (45.9)	507 (45.6)	1567 (46.5)
Women [*n* (%)]	685 (52.4)	516 (54.1)	604 (54.4)	1805 (53.5)
Persons/household (*n*)	3.9	4.3	4.5	4.2
Persons screened by Limoges questionnaire				
Persons screened [*n* (%)]	1077 (82.4)	757 (79.4)	847 (76.2)	2681 (79.5)
Persons with at least one positive answer (*n*)	60	58	50	168
Persons diagnosed with epilepsy (*n*)	39	48	41	128
Newly diagnosed [*n* (%)]	25 (64.1)	33 (68.7)	22 (53.7)	80 (62.5)
Previously diagnosed [*n* (%)]	14 (35.9)	15 (31.3)	19 (46.3)	48 (37.5)

Prevalence estimate	36.2/1000	63.4/1000	48.4/1000	47.7/1000
Confidence interval	25.8–49.5	46.8–84.1	34.7–65.7	39.8–56.8

Abbreviations: %, percentage; *n*, number.

### 3.2 Demographics of PwE

Striking differences in the demographics of PwE when analyzed by gender were observed (Table 2). The overall female:male ratio was 1.61:1.00. There was a male preponderance of PwE aged 9‐year and below inverting to a female preponderance in the age group 20‐years and above, also reflected in a large intergender difference in mean age. A new diagnosis of epilepsy was made in nearly 7 in 10 of all female PwE, compared to only 5 in 10 male patients (Table 3). In the age category of 30 years or older accounted 41.8% of females were newly diagnosed compared to only 24.5% of males.

**TABLE 2:** Demographics and medical history of PwE

**Demographics**	**Female**	**Male**	***p*‐value**	**Total**
Age (y) [*n*]	79	49		128
Mean age ± SD (y)	36.8 ± 19.5	20.2 ± 16.0	*p* < .001	30.4 ± 19.9
Median (y)	35.7	12.0		29.0
Minimum–maximum (y)	1.6–75.8	2.6–54.6		1.6–75.8

Age distribution [*n* (%)]	79 (100)	49 (100)	*p* < .001	128 (100)
0 to ≤9 y	7 (8.9)	20 (40.8)		27 (21.1)
10 to ≤19 y	9 (11.4)	9 (18.4)		18 (14.1)
20 to ≤29 y	18 (22.8)	5 (10.2)		23 (18.0)
30 to ≤39 y	16 (20.3)	7 (14.3)		23 (18.0)
≥40 y	29 (36.7)	8 (16.3)		37 (28.9)

Professional status [*n* (%)]	79 (100)	49 (100)	*p* = 0.003	128 (100)
Student	11 (13.9)	17 (34.7)		28 (21.9)
Farmer	46 (58.2)	10 (20.4)		56 (43.8)
Other	7 (8.9)	7 (14.3)		14 (10.9)
Unemployed	1 (1.3)	1 (2.0)		2 (1.6)
Missing	14 (17.7)	14 (28.6)		28 (21.9)

Concomitant conditions & medical history^a^ [*n* (%)]	60 (100)	31 (100)	NS	91 (100)
HIV positive	5 (8.3)	1 (3.2)		6 (6.6)
Cerebral malaria, meningitis	6 (10.0)	3 (9.7)		9 (9.9)
Tuberculosis	0 (0.0)	1 (3.2)		1 (1.1)
Diabetes mellitus, cardiovascular, respiratory	12 (20.0)	1 (3.2)		13 (14.3)
Headache/migraine	9 (15.0)	1 (3.2)		10 (11.0)
Mental health disorder	5 (8.3)	2 (6.5)		7 (7.7)
Other neurological conditions	6 (10.0)	7 (22.6)		13 (14.3)
Malnutrition	1 (1.7)	1 (3.2)		2 (2.2)
Head trauma	7 (11.7)	7 (22.6)		14 (15.4)
Birth trauma, perinatal asphyxia, cerebral palsy	2 (3.3)	5 (16.1)		7 (7.7)
Other	7 (1.7)	2 (6.5)		9 (9.9)

Family history epilepsy [*n* (%)]	79 (100)	49 (100)	NS	128 (100)
First‐degree relatives	12 (15.2)	9 (18.3)		21 (16.4)

Abbreviations: %, percentage; *n*, number; SD, standard deviation; y, year.

^a^ More than one concomitant medical condition could be reported per patient.

**TABLE 3:** Epilepsy characteristics by per gender

**Epilepsy characteristics**	**Female**	**Male**	***p*‐value**	**Total**
Diagnosis status [*n* (%)]	79 (100)	49 (100)	*p* < .05	128 (100)
Newly diagnosed epilepsy	55 (69.6)	25 (51.0)		80 (62.5)
Previously diagnosed epilepsy	24 (30.4)	24 (49.0)		48 (37.5)

Age of onset of epilepsy [*n* (%)]^a^	70 (100)	46 (100)	*p* < .001	116
Mean ± SD (y)	27.1 ± 19.5	13.2 ± 14.2		21.6 ± 18.8
Median (y)	25.3	7.7		16.3
Minimum–maximum (week‐year)	1 w–70.8 y	4 w–54.2 y		1 w–70.8 y

Time since first seizure [*n*]^a^	70	46	NS	116
Mean ± SD (y)	9.8 ± 12.2	7.3 ± 7.8		8.8 ± 10.7
Median (y)	5.0	4.4		5.0
Minimum–maximum (week‐year)	2 w–67.1 y	0 w–32.0 y		0 w–67.1 y

Age of onset of epilepsy, newly diagnosed only [*n*]^a^	50	24	*p* < .05	73
Mean ± SD (y)	28.64 ± 20.6	17.7 ± 15.8		24.5 ± 19.1
Median (y)	26.5	12.7		23.7
Minimum–maximum	1 y–70.8 y	1 w–54.2 y		1 w–66.8 y

Time since first seizure, newly diagnosed only [*n*]^a^	50	24	NS	73
Mean ± SD (y)	9.08 ± 12.6	6.4 ± 7.4		8.3 ± 11.3
Median (y)	5.0	3.6		5.0
Minimum–maximum (week‐year)	6 w–67.1 y	6 w–32.0 y		6 w–67.1 y

Seizure classification [*n* (%)]	79 (100)	49 (100)	NS	128 (100)
Focal	47 (59.5)	22 (44.9)		69 (53.9)
Focal to bilateral	21 (44.7)	13 (59.1)		34 (49.3)
Motor, normal/impaired awareness	12 (25.5)	3 (13.6)		15 (21.7)
Non‐motor, normal/impaired awareness	14 (29.2)	6 (27.3)		20 (29.0)
Generalized	2 (2.5)	2 (4.1)		4 (3.1)
Unknown	26 (32.9)	24 (49.0)		50 (39.1)
Motor	18 (69.2)	16 (66.6)		34 (68.0)
Non‐motor	8 (30.1)	8 (33.3)		16 (32.0)
Unclassified	4 (5.1)	1 (2.0)		5 (3.9)

Seizure frequency per month [*n* (%)]^a^	72 (100)	42 (100)	NS	114(100)
0	1 (1.4)	4 (9.5)		5 (4.4)
1 to ≤2	27 (37.5)	21 (50.0)		48 (42.1)
3 to ≤5	15 (20.8)	5 (11.9)		20 (17.5)
6 to ≤29	13 (18.1)	7 (16.7)		20 (17.5)
≥30	16 (22.2)	5 (11.9)		21 (18.4)

Clinical examination [*n* (%)]	79 (100)	49 (100)	NS	128 (100)
Normal	81 (77.2)	38 (77.6)		99 (77.3)
Pregnancy	2			2
Abnormal	18 (22.8)	11 (22.4)		29 (22.7)
Agitation	0 (0.0)	2 (4.1)		2 (1.6)
Burns	4 (5.1)	2 (4.1)		6 (4.7)
Confusion	2 (2.5)	2 (4.1)		4 (3.1)
Dysmorphia	3 (3.8)	0 (0.0)		3 (2.3)
Neurological signs	5 (6.3)	5 (10.2)		10 (7.8)
Others	4 (5.1)	0 (0.0)		4 (3.1)

Abbreviations: *n*, number; NS, not significant; SD, standard deviation; w, week; y, year.

^a^ Age first seizure and seizure frequency not documented in several patients.

Concomitant infectious diseases, including tuberculosis and malaria, were reported equally in 18.3% and 16.1% of female and male PwE, respectively. HIV status was not available for 53 (41.4%) PwE and 6 were HIV positive. Head trauma and birth trauma, asphyxia, and cerebral palsy were more frequently reported in male PwE. Sporadic use of recreational drugs or alcohol was reported by 21 (16.4%) PwE.

First‐degree and second‐degree family history of epilepsy was reported by 21 (16.4%) and 13 (10.2%) PwE, respectively.

### 3.3 Epilepsy characteristics

Age at onset of first seizure differed markedly between male and female PwE. Comparing newly diagnosed to previously diagnosed PwE, a trend of epilepsy onset at an older age for newly diagnosed female PwE was observed (Tables S1 and S2).

The median time since first seizure to diagnosis for newly diagnosed PwE was 5 years, with 19.0% being diagnosed within a year and another 34.2% within 5 years after first seizure. Due to missing data of exact date of diagnosis, time to diagnosis for previously diagnosed PwE could not be estimated. Male PwE were more frequently diagnosed with epilepsy at younger age, with 82.6% was less than 20 years old versus 47.6% of previously diagnosed female PwE.

Five PwE, of whom four previously diagnosed, reported seizure freedom longer than 12 months, including four longer than two and one longer than 5 years. Seizure frequency up to maximum two per month, was reported by 42.1%. Of note, 18.4% reported 30 or more seizures per month. No status epilepticus was reported.

Based on the 2017 International League Against Epilepsy (ILAE) seizure classification, a focal‐onset seizure was reported in 69 (53.9%), 34 of whom had focal to bilateral seizures. Generalized and unknown onset was observed in 4 (3.1%) and 50 (39.1%) of PwE respectively. One patient had typical absence epilepsy. Focal seizures were reported more frequently in newly diagnosed PwE. Diagnosis was relying heavily on detailed seizure history as EEG and CT had been performed in only 5.5% and 3.1% of PwE respectively. Seizure onset and classification were similar for male and female PwE.

The clinical examination was normal in approximately four out of five PwE. Burns were equally observed in men and women in one in 20. Six persons reported agitation, confusion, and other neurological symptoms, for example, dyskinesia, jerky movements, spastic hypertonia, motor development retardation, were observed in 10 PwE. There were no significant gender differences. Two female PwE were pregnant.

### 3.4 Treatment

Of 48 previously diagnosed PwE, 11/48 (22.9%) were on treatment, resulting in a treatment gap of 77.1%. In contrast, five out of six (83.3%) HIV positive PwE had continued their anti‐retroviral treatment. Previous or ongoing AED monotherapy was reported or recalled in 90.0% PwE and combination therapy in four 10.0%. Phenobarbital, carbamazepine, sodium valproate were mentioned each by 10% or more of previously diagnosed PWE.

**FIGURE 2:**
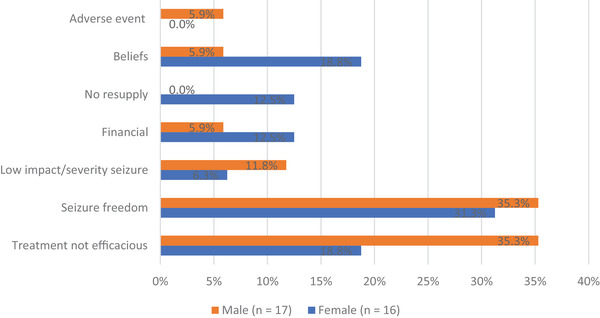
Reasons (in %) for treatment discontinuation in previously diagnosed PwE by gender

Reasons for discontinuation of any AED treatment were cited by 33, of whom 16 female patients: seizure cessation in 11/33 (33.3%), lack of efficacy in 10 (30.3%) and beliefs such as “epilepsy is untreatable” in 3 (9.1%) PwE. Of 11 patients citing seizure cessation as reason for discontinuation, only two were actually seizure‐free for more than a year upon clinical exam whilst others had ongoing seizures. Gender differences for reason of discontinuation between men and women included beliefs and ignorance more frequently cited by female PwE compared to low seizure impact and perceived AED inefficacy by males (Figure 2).

### 3.5 Access to healthcare and care seeking pattern

Medical insurance coverage was available for 103/128 (80.5%) PwE, equal for female and male PwE at 79.7% and 81.6%, respectively.

**FIGURE 3:**
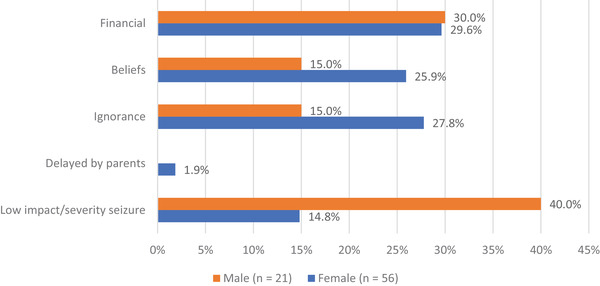
Reasons (in %) for delaying biomedical care treatment seeking in newly diagnosed PwE by gender (*n* = number reasons mentioned)

Four newly diagnosed PwE, of whom 3 women, had sought biomedical care and had been misdiagnosed. Of 67 (83.8%) remaining newly diagnosed PwE reporting reason for delay to seek biomedical care, 47 female and 20 male PwE reported 56 and 21 reasons, respectively. Five main categories of the 77 reasons were identified: financial constraints, ignorance, (personal and family) beliefs, low impact or low severity of seizures, and misdiagnosis (Figure 3). Up to 30% reported financial constraints as reason to postpone seeking care. In addition, female PwE tended to delay seeking care due to beliefs and ignorance, whereas men tended to delay due to a low impact of the condition on their daily life.

Of all PwE, 22 (17.2%) had contacted traditional or faith healers, of whom 14 (10.9%) PwE without seeking biomedical care and 8 (6.3%) PwE combining traditional medicine with biomedical care. Men (59.1%) more frequently used traditional practices than women (40.9%).

### 3.6 Gender differences—Literature review

Our literature search resulted in 39 manuscripts of which eight had a door‐to‐door design and reported gender‐specific prevalence of lifetime and active epilepsy.

One manuscript reported prevalence in five distinct sub Saharan regions, represented separately in Table 4. Male/female prevalence in different studies was highly variable, ranging from 3.2 to 123‰ for males and 2.5 to 90‰ for females. Also, gender ratio varied markedly. Of note are the differences in study design, case definitions, and study populations, for example, age cut‐off, active versus lifetime epilepsy, and adjusted versus crude prevalence rate. All studies, with an exception of the Benin study, were conducted in rural areas.

**TABLE 4:** Prevalence of epilepsy in D2D surveys in sub‐Saharan Africa

**Country**	**Study year**	**Sample size**	**Active/lifetime epilepsy**	**Prevalence**	**Age limit (y)**	**PwE#F/#M**	**PwE F/M ratio**	**Prevalence male (‰)**	**Prevalence female (‰)**
Benin–Cotonou	2012	13,046	Active < 5 y	Crude	≥15	51/54	0.94:1.00	9.8	6.8
Ghana–Kintampo	2013	129,812	Active < 1 y	Age‐adjusted	All	ND	ND	11.1	9.1
Cameroon–Keleng	2008	181	Active < 5 y	Age‐adjusted	All	9/10	0.90:1.00	123	90
Central African Republic	2015	1023	Lifetime	Crude	All	6/6	1.00:1.00	13.0	10.7
Kenya	2003	151,408	Active < 1 y	Crude	≥6	220/225	0.97:1.00	3.2	2.7
Kenya–Kilifi	2007–2008	233,881	Active < 1 y	Age‐adjusted	All	ND	0.92:1.00	7.0	8.6
Tanzania–Hai	2009–2010	103,026	Active < 5 y	Crude	≥15	136/155	0.91:1.00	3.2	2.5
Tanzania	1999	4905	Active < 5 y	Age‐adjusted	All	21/21	1.00:1.00	7.5	7.2
Tanzania	2009	7399	Lifetime	Crude	All	52/31	1.46:1.00	8.4	14.3
Tanzania–Ifakara	2009	93,645	Active < 1 y	Age‐adjusted	All	ND	ND	14.0	10.9
Uganda–Iganga‐Mayuge	2009	64,172	Active < 1 y	Age‐adjusted	All	ND	ND	8.9	9.6
South Africa–Agincourt	2008–2009	82,818	Active < 1 y	Age‐adjusted	All	ND	ND	6.6	7.6

Abbreviations: #, number; ‰, per thousand; F, female; M, male; ND, not documented; y, year.

## 4 DISCUSSION

### 4.1 Prevalence and gender

Prevalence of epilepsy and characteristics of PwE from door‐to‐door survey in rural Rwanda were reported. At 47.7‰ (CI 39.8–56.8), the point prevalence remained unchanged over time compared to previously reported prevalence of 49.0‰ from rural and urban areas in 2005 (Sebera et al., 2015). Prevalence varied across villages, yet this was not statistically significant.

This prevalence of lifetime epilepsy is high compared to a recently published meta‐analysis on prevalence of (lifetime and active) epilepsy in SSA, with an overall 9.6‰ and 4.8‰ prevalence in rural and urban settlements, respectively (Owolabi et al., 2020). Our prevalence is even higher than the highest prevalence of 30.2‰ in Central African countries (Owolabi et al., 2020). It is also higher than previously reported in Rwanda using a one epilepsy question screening tool in a cross‐sectional study (Simms et al., 2008). It is considered that cross‐sectional studies may underestimate the prevalence of lifetime epilepsy by 75%, and of non‐convulsive epilepsy by up to 50% (Ngugi et al., 2013). We deem it unlikely that our data largely underestimate prevalence in this region given the good sensitivity of the Limoges questionnaire as previously demonstrated and as we identified 37 in 128 (28.9%) PwE with non‐motor seizures, which are difficult to identify. In addition, the positive predictive value of our screening was similar to other door‐to‐door surveys (Dent et al., 2005; Ndoye et al., 2005; Njamnshi et al., 2008).

In our cohort, a significant gender difference with high number of total and newly diagnosed female PwE was observed. In SSA, reported gender‐specific prevalence of epilepsy is highly variable (Table 4), with higher male or with higher female prevalence across age groups and geographies. Our results illustrate a female:male ratio which inverted with age, exceptionally low at 0.35:1.00 in the 9 years or below age group and high at 3.60:1.00 in the age group above 40 years‐of‐age. This age‐specific prevalence inversion has been observed in other SSA countries with a male predominance in the age group < 15 years reported in several door‐to‐door and hospital‐based surveys (Atugonza et al., 2016; Bistervels et al., 2016; Lagunju et al., 2016; Matonda‐Ma‐Nzuzi et al., 2019). One study reported a female:male ratio as low as 0.64:1.00 (Hunter et al., 2012). The female:male ratio of 3.60:1.00 in PwE aged 40 years and above is markedly higher when compared to other surveys with a high female:male ratio of up to 2.00:1.00 (Birbeck & Kalichi, 2004; Coleman et al., 2002; Ezeala‐Adikaibe et al., 2016; Mwangala et al., 2018). Our observations prove consistent with age of onset of first seizure when comparing all and newly diagnosed PwE, with possible explanations, including age‐specific aetiologies, care seeking patterns, degree of self‐care, gender‐dependent risk factors, competing mortality risks, or even a study bias due to case ascertainment (Birbeck & Kalichi, 2004; Winkler et al., 2009).

### 4.2 Epilepsy characteristics and gender specifics

Possible gender‐specific factors relative to aetiology and epilepsy characteristics were further explored.

Seizure‐onset classification according to the ILAE 2017 guidelines nor seizure frequency were different between sexes. Age of first seizure differed in men versus women, probably only reflecting observed differences in age group distribution.

Head injuries, up to 15.4% of all reported comorbidities, were reported by 14.3% (7/49) of male PwE. This is in line with 15.2% of PwE more than 18 y reporting head injuries in Nigeria, yet high considering 60% of our male population was less than 20 y (Lagunju et al., 2016). In this age group, frequencies of head injury varied from 3.6%, 3.9–4% to 8–8.8% (Burton et al., 2012; Kakooza‐Mwesige et al., 2017; Ngugi et al., 2013), suggesting a trend towards increased number of head injuries with age.

The factor of having a first‐degree relative living with epilepsy did not differ by gender at 14% and is in line with 11.6%, 13%, and 14.5% reported in Tanzania, South Africa, and Uganda, respectively (Ackermann et al., 2019; Burton et al., 2012; Kakooza‐Mwesige et al., 2017).

Co‐morbidity or history of infectious diseases affecting more than 10%, was similar for both genders and in line or slightly lower (range 5.5%–13%) than in other SSA regions (Assadeck et al., 2019; Bistervels et al., 2016; Burton et al., 2012; Carter et al., 2004; Kariuki et al., 2014; Samia et al., 2019). The frequency of cerebral malaria in our cohort of PwE was higher than the reported malaria prevalence of 1% in the general population in the Northern Province in Rwanda (Malaria and Other Parasitic Diseases Division of the Rwanda Biomedical Center & ICF, 2018).

There is a clear association between epilepsy and HIV with new‐onset seizures occurring in up to 11% of HIV infected persons (Howlett, 2019). The observed 4.6% of a HIV positive status was somewhat higher than the percentage of 1.7% observed in a Rwandan tertiary epilepsy center, and higher than the 2.6% of HIV positive PwE in the province (Nsanzimana et al., 2017; Van Steenkiste et al., 2019). It is noteworthy that no case of neurocysticercosis was reported, possibly due to low number of imaging studies and unavailability of diagnostic serological tests.

The relationship between epilepsy and perinatal complications, such as prolonged labor and birth asphyxia, has clearly been acknowledged (Osakwe et al., 2014). Five of 7 patients reporting perinatal complications, were male and accounted for more than 10% of male PwE. Total frequency of 5.5% was also higher than elsewhere reported (Osakwe et al., 2014).

### 4.3 Access to medical care, care seeking patterns, and gender

The diagnosis gap was 62.5% with a 68.4% gap for female PwE and 51.0% for male PwE. We observed an overall treatment gap of 91.5% (117/128).

Financial reasons and access to care were not associated with gender differences, with a same percentage in men and women. Financial reasons were also evenly cited by both groups for either discontinuing treatment as for not seeking care. Professional status differed with more female reporting farmer as occupation, explained as an artifact given the age group distribution. Indeed, of all PwE above 20 y of age, about 15% of male and female reported an inactive or missing professional status. We did not document monthly income of PwE which may be a confounding factor. Yet, in the rural regions in Rwanda, most households are in the lowest economic class. As the three screened villages had a similar rural setting, we consider a bias due to economic status unlikely for diagnosis and treatment gap. We cannot exclude however that specific roles in the household, for example owning the financial budget, affect care seeking patterns. Geography itself has been reported to influence care seeking patterns, with patient reported proximity to the biomedical care center as a determining factor (Liu et al., 2019; Rutebemberwa et al., 2020). Our data did not allow an ascertainment of geographical distance as a determinant for gender differences.

For both diagnosis and treatment gap, a trend for gender differences was observed with female PwE citing beliefs and ignorance more frequently compared to male PwE. The latter mentioned more seizure and treatment related reasons. This observation was consistent between newly diagnosed and previously diagnosed patients. In our cohort, 22 PwE sought *care* with traditional healers or faith healers, of whom more than half did not seek biomedical care. Interestingly, this was more prevalent in male PwE, which has not been reported previously.

Proposed solutions to address the diagnosis and treatment gap, include involvement of CHWs to promote epilepsy care, social reconnection, and mutual support for PwE within communities (Mottiar & Lodge, 2018; Santos et al., 2019). CHWs are fully integrated and respected influencers in the village's core community activities. Second, social support groups play a vital role enabling community members to own their community healing practices, while maintaining existing social networks. Community's healing practices include initiatives, such as *umusabane* (social party for sharing life), *umuganda* (collective work to help vulnerable members), and *umubugizi* (mediation of reconciliation) (Otake, 2018). In our cohort, CHWs accompanied persons to the HC, and they stayed during the clinical investigation at the side of the positively screened person. Following the diagnosis and discussion of the proposed treatment, PwE returned with the CHWs in their communities. Under supervision of the CHW an early integration into the family and community was envisioned.

### 4.4 Limitations of this study

Incomplete screening of all villagers may induce an enrolment bias geared towards villagers staying at home, for example, children and women. Notwithstanding repeat visits, only 80% of villagers were screened, slightly lower than screening rates of 87% reported in a three‐staged study design (Ngugi et al., 2013). Our distribution of screened villagers with 56% women reflected closely the census data with 54% female citizens and therefore our screening process is unlikely to have resulted in a bias towards gender or age overrepresentation. The gap of 20% does require an optimization of future screening processes. We cannot exclude a sample bias due to village selection, yet no significant differences in prevalence were observed.

Some of the observed differences from co‐morbidities and epilepsy characteristics may be age‐or gender‐specific and therefore our observations must be interpreted with caution and considered as hypothesis generating only. We recommend future studies to include possible gender differences relative to age groups, aetiology, economic and sociocultural aspects.

## 5 CONCLUSION

We confirmed a high prevalence of epilepsy in rural regions in Rwanda of 47.7‰. The observed female/male ratio in PwE is the highest of any published door‐to‐door survey in SSA. Further research is warranted to understand whether the repeatedly observed gender divergences represent differences in aetiology, care seeking patterns, gender‐specific risk factors, a sample/study bias, or even competing mortality risks. To address the variability of prevalence across and possibly within countries, coordinated multi‐country research efforts with an emphasis on etiology are necessary, including sound sample sizes, screening, and diagnostic protocols, and integration with other demographic, epidemiological, economic, and cultural datasets. With a diagnosis gap of 62.5% and a treatment gap of 91.4%, our data reiterate the need to better understand the drivers of care seeking patterns and the need to adapt epilepsy public health strategies towards gender‐specific needs related to education of PwE and communities, with a probably vital role for CHWs (Anand et al., 2019; Matonda‐Ma‐Nzuzi et al., 2019; Mottiar & Lodge, 2018; Tuyisenge et al., 2020).

## CONFLICT OF INTEREST

Travel and lodging for the senior resident‐neurologist were provided by the Department of Neurology, Ghent University. Dirk E. Teuwen is a consultant of UCB Pharma. Peter Dedeken received consultancy fees from UCB Pharma, Merck, and Novartis. Paul A. J. M. Boon received speaker and consultancy fees from UCB Pharma, LivaNova, and Medtronic, and research grants from the same companies through his institution. Other authors have no competing interests.

### PEER REVIEW

The peer review history for this article is available at https://publons.com/publon/10.1002/brb3.2377

## Supporting information

Supporting information

## Data Availability

The data that support the findings of this study are available on request from the corresponding author. The data are not publicly available due to privacy or ethical restrictions.
