# Supplementary material for: High prevalence of epilepsy in Northern Rwanda: Exploring gender differences
Source: Brain Behav. 2021 Oct 17;11(11):e2377. doi: 10.1002/brb3.2377 (PMC8613444; doi:10.1002/brb3.2377)
Supplement: Supplementary file 1 — Supporting information [file BRB3-11-e2377-s001.docx]

**Supplementary Tables: Newly *vs.* previously diagnosed PwE**

| **Demographics** | **Newly diagnosed** | **Previously diagnosed** | **p-value** | **Total** |
| --- | --- | --- | --- | --- |
| All PwE | 80 (100) | 48 (100) |  | 128 (100) |
| Age |  |  | NS |  |
| Mean age ± SD | 33.2 ± 20.7 | 25.9 ± 17.9 |  | 30.4 ± 19.9 |
| Median | 32.7 | 25.0 |  | 29.0 |
| Age distribution all PwE [n (%)] |  |  | NS |  |
| 0y – ≤ 9y | 16 (20) | 11 (22.9) |  | 27 (21.1) |
| 10y – ≤ 19y | 9 (11.3) | 9 (18.8) |  | 18 (14,1) |
| 20y – ≤ 29y | 10 (12.5) | 13 (27.1) |  | 23 (18.0) |
| 30y – ≤ 39y | 17 (21.3) | 6 (12.5) |  | 23 (18.0) |
| ≥ 40y | 28 (35) | 9 (18.8) |  | 37 (28.9) |
| Female PwE | 55 (100) | 24 (100) | NS | 79 (100) |
| Age (female PwE) |  |  |  |  |
| Mean age ± SD | 39.5 ± 20.6 | 33.1 ± 16.1 |  | 36.8 ± 19.5 |
| Median | 37.1 | 28.3 |  | 35,7 |
| Age distribution female PwE [n (%)] |  |  | NS |  |
| 0y – ≤ 9y | 5 (9.1) | 2 (8.3) |  | 7 (8.9) |
| 10y – ≤ 19y | 7 (12.7) | 2 (8.3) |  | 9 (11.4) |
| 20y – ≤ 29y | 9 (16.4) | 9 (37.5) |  | 18 (22.8) |
| 30y – ≤ 39y | 12 (21.8) | 4 (16.7) |  | 16 (20.3) |
| ≥ 40y | 22 (40.0) | 7 (29.2) |  | 29 (36.7) |
| Male PwE | 25 (100) | 24 (100) | NS | 49 (100) |
| Age (male PwE) |  |  |  |  |
| Mean age ± SD | 23.2 ± 17.9 | 16.7 ± 13.2 |  | 20.2 ± 16.0 |
| Median | 13.1 | 12.0 |  | 12.0 |
| Age distribution male PwE [n (%)] |  |  | NS |  |
| 0y – ≤ 9y | 11 (44.0) | 9 (37.5) |  | 20 (40.8) |
| 10y – ≤ 19y | 2 (8.0) | 7 (29.2) |  | 9 (18.4) |
| 20y – ≤ 29y | 1 (4.0) | 4 (16.7) |  | 5 (10.2) |
| 30y – ≤ 39y | 5 (20.0) | 2 (8.5) |  | 7 (14.3) |
| ≥ 40y | 6 (24.0) | 2 (8.5) |  | 8 (16.3) |
|  |  |  |  |  |
| Professional status [n (%)] | 80 (100) | 48 (100) | NS | 128 (100) |
| Student | 14 (17.5) | 14 (29.2) |  | 28 (21.9) |
| Farmer | 39 (48.8) | 17 (35.4) |  | 56 (43.8) |
| Other | 11 (13.8) | 3 (6.3) |  | 14 (10.9) |
| Unemployed | 1 (1.3) | 1 (2.1) |  | 2 (1.6) |
| Missing | 15 (18.8) | 13 (27.1) |  | 28 (21.9) |
| Concomitant conditions & medical history^a^ [n (%)] | 58 (100) | 33 (100) | NS | 91 (100) |
| HIV positive | 4 (6.9) | 2 (6.1) |  | 6 (6.6) |
| Cerebral malaria, meningitis | 6 (10.3) | 3 (9.1) |  | 9 (9.9) |
| Tuberculosis | 1 (1.7) | 0 (0.0) |  | 1 (1.1) |
| Diabetes mellitus, cardiovascular, respiratory | 10 (17.2) | 3 (9.1) |  | 13 (14.3) |
| Headache/migraine | 7 (12.1) | 3 (9.1) |  | 10 (11.0) |
| Mental health disorder | 3 (5.2) | 4 (12.1) |  | 7 (7.7) |
| Other neurological conditions | 6 (10.3) | 7 (21.6) |  | 13 (14.3) |
| Malnutrition | 1 (1.7) | 1 (3.0) |  | 2 (2.2) |
| Head trauma | 8 (13.8) | 6 (18.2) |  | 14 (15.4) |
| Birth trauma, perinatal asphyxia, cerebral palsy | 4 (6.9) | 3 (9.1) |  | 7 (7.7) |
| Other | 8 (13.8) | 1 (3.0) |  | 9 (9.9) |
| Family history epilepsy [n (%)] | 80 (100) | 48 (100) | NS | 128 (100) |
| First degree relatives | 15 (18.8) | 6 (12.86) |  | 21 (16.4) |

**Table S-1**. Demographics and medical history of PwE by newly or previously diagnosed. Abbreviations: (^a^) more than one concomitant medical condition could be reported per patient; n: number; %: percentage; SD: standard deviation; y: year

| **Epilepsy characteristics** | **Newly diagnosed** | **Previously diagnosed** | **p-value** | **Total** |
| --- | --- | --- | --- | --- |
| **All PwE** |  |  |  |  |
| Diagnosis status [n (%)] | 80 (100) | 48 (100) | p<0.05 | 128 (100) |
| Female PwE | 55 (68.8) | 24 (50.0) |  | 79 (61.7) |
| Male PwE | 25 (31.2) | 24 (50.0) |  | 49 (38.3) |
| Age of onset of epilepsy [n (%)]^a^ | 73 (100) | 43 (100) | p < 0.001 | 116 (100) |
| Mean ± SD (y) | 24.5 ± 19.1 | 16.7 ± 17.4 |  | 21.6 ± 18.8 |
| Median (y) | 23.7 | 12.0 |  | 16.3 |
| Minimum – maximum | 1w – 70.8y | 4w – 54.2y |  | 1w – 70.8y |
| Time since first seizure [n (%)]^a^ | 73 (100) | 43 (100) | NS | 116 (100) |
| Mean ± SD (y) | 8.3 ± 11.3 | 9.8 ± 9.7 |  | 8.8 ± 10.7 |
| Median (y) | 5.0 | 6.0 |  | 5.0 |
| Minimum – maximum | 2w – 67.1y | 0w – 34.0y |  | 0w – 67.1y |
| **Female PwE** |  |  |  |  |
| Age of onset of epilepsy [n (%)]^a^ | 50 | 20 | p<0.05 | 70 |
| Mean ± SD (y) | 28.6 ± 20.6 | 23.4 ± 16.4 |  | 27.1 ± 19.5 |
| Median (y) | 26.5 | 20.5 |  | 25.3 |
| Time since first seizure [n (%)]^a^ | 50 | 20 | NS | 70 |
| Mean ± SD (y) | 9.07 ± 12.6 | 11.7 ± 11.0 |  | 9.8 ± 12.2 |
| Median (y) | 5.0 | 7.9 |  | 5.0 |
| **Male PwE** |  |  |  |  |
| Age of onset of epilepsy [n (%)]^a^ | 24 | 22 | p = 0.03 | 46 |
| Mean ± SD (y) | 17.7 ± 15.8 | 8.2 ± 10.3 |  | 13.2 ± 14.2 |
| Median (y) | 12.7 | 4.4 |  | 7.7 |
| Time since first seizure, male [n (%)]^a^ | 24 | 22 | NS | 46 |
| Mean ± SD (y) | 6.4 ± 7.4 | 8.4 ± 8.3 |  | 7.3 ± 7.8 |
| Median (y) | 3.6 | 5.9 |  | 4.4 |
|  |  |  |  |  |
| Seizure classification [n (%)] | 80 (100) | 48 (100) | NS | 128 (100) |
| Focal | 50 (62.5) | 19 (49.6) |  | 69 (53.9) |
| *Focal to bilateral* | *19 (38)* | *15 (78.9)* |  | *34 (49.3)* |
| *Motor, normal/impaired awareness* | *12 (24)* | *3 (15.8)* |  | *15 (21.7)* |
| *Non-motor, normal/impaired awareness* | *19 (38)* | *1 (5.3)* |  | *20 (29.0)* |
| Generalized | 3 (3.8) | 1 (2.1) |  | 4 (3.1) |
| Unknown | 23 (28.8) | 27 (56.3) |  | 50 (39.1) |
| *Motor* | *13 (16.5)* | *21 (42.9)* |  | *34 (68.0)* |
| *Non-motor* | *10 (12.7)* | *6 (12.2)* |  | *16 (32.0)* |
| Unclassified | 4 (5.0) | 1 (2.1) |  | 5 (3.9) |
| Seizure frequency per month [n (%)]^a^ | 71 (100) | 43 (100) | NS | 114(100) |
| 0 | 1 (1.4) | 4 (9.3) |  | 5 (4.4) |
| 1 to ≤ 2 | 27 (38.0) | 21 (48.8) |  | 48 (42.1) |
| 3 to ≤ 5 | 16 (22.5) | 4 (9.3) |  | 20 (17.5) |
| 6 to ≤ 29 | 13 (18.3) | 7 (16.3) |  | 20 (17.5) |
| ≥ 30 | 14 (19.7) | 7 (156.3) |  | 21 (18.4) |
| Clinical examination [n (%)] | 80 (100) | 48 (100) | NS | 128 (100) |
| Normal | 68 (85.0) | 31 (64.6) |  | 99 (77.3) |
| *Pregnancy* | 1 | 1 |  | 2 |
| Abnormal | 12 (15.0) | 17 (35.4) |  | 29 (22.7) |
| *Agitation* | 0 (0.0) | 2 (4.1) |  | 2 (1.6) |
| *Burns* | 2 (2.5) | 4 (8.2) |  | 6 (4.7) |
| *Confusion* | 0 (0.0) | 4 (8.2) |  | 4 (3.1) |
| *Dysmorphia* | 1 (1.3) | 2 (4.1) |  | 3 (2.3) |
| *Neurological signs* | 5 (6.3) | 5 (10.2) |  | 10 (7.8) |
| *Others* | 4 (5.1) | 0 (0.0) |  | 4 (3.1) |

**Table S-2**. Epilepsy characteristics by gender by newly and previously diagnosed Abbreviations: (^a^) age first seizure and seizure frequency not documented in several patients; n: number, w: week; y: year; NS = not significant; SD: Standard Deviation
